# A rare case of aortic dissection presenting as pure transient global amnesia

**DOI:** 10.5830/CVJA-2015-061

**Published:** 2015

**Authors:** Hirsh Kaveeshvar, Rabih Kashouty, Vivek Loomba, Noor Yono

**Affiliations:** Department of Neurology, Henry Ford Hospital, Detroit, USA; Department of Neurology, Icahn School of Medicine, Mount Sinai Beth Israel, New York, USA; Department of Anaethesiology and Pain Medicine, Henry Ford Hospital, Detroit, USA; Department of Neurology, North Shore University Hospital, Hofstra University, New York, USA

**Keywords:** transient global amnesia, aortic dissection, TIA, emergency, Valsalva

## Abstract

Transient global amnesia (TGA) is a well-described neurological phenomenon. Clinically, it manifests with the sudden onset of a paroxysmal, transient loss of anterograde memory and disorientation but with intact consciousness. Typically, symptoms last for only a few hours. We present an unusual case of aortic dissection presenting with pure TGA in a patient, who had a positive outcome. This is the second case report of a patient with aortic dissection presenting with pure TGA syndrome, but it is the first case in which the patient survived.

## Abstract

Transient global amnesia (TGA) is a well-described neurological phenomenon. Clinically, it manifests with a sudden onset of a paroxysmal, transient loss of anterograde memory and disorientation but with intact consciousness.^1^ Typically symptoms last for only a few hours. The aetiology of TGA remains unclear.^1^,^2^ Few case reports have described a link between TGA and aortic dissection (AD). We present an unusual case of AD presenting with pure TGA in a patient who had a positive outcome.

## Case report

A 63-year-old man with a history of hypertension and hyperlipidaemia suddenly developed anterograde and retrograde amnesia and was admitted to our hospital. The patient, who was in the passenger seat of a car, with his colleague driving, suddenly became pale and dizzy and was not aware of his surroundings. He repeatedly asked the reason they were in the car. The patient denied any complaints, including chest pain, but asked repetitive questions.

On general examination, the patient was afebrile. His blood pressure was 94/53 mmHg. His neurological examination was unremarkable, except for anterograde amnesia. Blood tests revealed mild leukocytosis (15 700 cells/mm^3^). A chest X-ray showed no abnormalities, a head computed tomography was unremarkable, and an electroencephalography revealed no epileptiform discharges.

The anterograde amnesia resolved 10 hours after onset. However, he remained hypotensive and a mild diastolic murmur was noted over the aorta. Urgent cardiac echocardiography revealed Stanford type A AD. The patient was immediately taken to the operating room and successfully rescued. He was discharged without any significant neurological symptoms.

## Discussion

This case illustrates an example of painless AD presenting with pure TGA with no focal neurological deficits. The persistence of hypotension and the presence of an aortic murmur after the resolution of TGA raised the suspicion of AD.

The aetiology of TGA remains unclear.^1^,^2^ Traditionally, it is believed to be due to transient cerebral ischaemia, particularly in the hippocampal formation and mesiotemporal structures; however, evidence is now accumulating that fails to show diffusion-weighted imaging hyperintensities within 24 hours of onset of symptoms.^1^,^3^

AD is a life-threatening emergency and prompt clinical recognition is essential for treatment.^4^ Acute neurological syndromes in AD are uncommon and typically present with focal neurological deficits.^5^ It can mimic a large group of neurological symptoms, including TGA, despite the absence of chest pain.^6^ In a series of 977 patients, Park *et al.*^7^ observed only 63 (6.4%) patients with painless AD, which may mislead the physician and delay the treatment. The existence of a pathogenic link between pure TGA and AD is still unclear.^8^

Transient episodes of increased intrathoracic pressure can potentially precipitate AD.^9^ Similarly, transient episodes of increased intrathoracic pressure due to the Valsalva manoeuvre have been noted to be a precipitating factor of TGA. Ultrasound studies during the Valsalva manoeuvre demonstrate a reduction in lumen diameter of the superior vena cava due to increased intrathoracic pressure, which causes obstruction of the venous blood flow transmitting venous back pressure towards the brain.^10^ While the association between AD and TGA remains unclear, the shared common precipitant of raised intrathoracic pressure may provide a link between the two processes.

## Conclusion

This report emphasises the importance of detailed physical examination in patients with pure TGA. Physicians should be aware of such a possibility and suspect AD in patients with pure TGA.

To our knowledge, this is the second case report of a patient with AD presenting with pure TGA syndrome.^10^ In the only other case reported, the patient had a fatal outcome. This is therefore the first case report of a patient surviving after admission with AD presenting as pure TGA syndrome.
